# Ecological Stoichiometry beyond Redfield: An Ionomic Perspective on Elemental Homeostasis

**DOI:** 10.3389/fmicb.2017.00722

**Published:** 2017-04-25

**Authors:** Punidan D. Jeyasingh, Jared M. Goos, Seth K. Thompson, Casey M. Godwin, James B. Cotner

**Affiliations:** ^1^Department of Integrative Biology, Oklahoma State UniversityStillwater, OK, USA; ^2^Department of Biology, University of Texas at ArlingtonArlington, TX, USA; ^3^Water Resources Science Program, University of MinnesotaSt. Paul, MN, USA; ^4^School of Natural Resources and Environment, University of MichiganAnn Arbor, MI, USA; ^5^Department of Ecology, Evolution, and Behavior, University of MinnesotaSt. Paul, MN, USA

**Keywords:** elemental profiling, freshwater heterotrophic bacteria, ionome, ionomics, nutrient limitation, phosphorus supply

## Abstract

Elemental homeostasis has been largely characterized using three important elements that were part of the Redfield ratio (i.e., carbon: nitrogen: phosphorus). These efforts have revealed substantial diversity in homeostasis among taxonomic groups and even within populations. Understanding the evolutionary basis, and ecological consequences of such diversity is a central challenge. Here, we propose that a more complete understanding of homeostasis necessitates the consideration of other elements beyond C, N, and P. Specifically, we posit that physiological complexity underlying maintenance of elemental homeostasis along a single elemental axis impacts processing of other elements, thus altering elemental homeostasis along other axes. Indeed, transcriptomic studies in a wide variety of organisms have found that individuals differentially express significant proportions of the genome in response to variability in supply stoichiometry in order to maintain varying levels of homeostasis. We review the literature from the emergent field of ionomics that has established the consequences of such physiological trade-offs on the content of the entire suite of elements in an individual. Further, we present experimental data on bacteria exhibiting divergent phosphorus homeostasis phenotypes demonstrating the fundamental interconnectedness among elemental quotas. These observations suggest that physiological adjustments can lead to unexpected patterns in biomass stoichiometry, such as correlated changes among suites of non-limiting microelements in response to limitation by macroelements. Including the entire suite of elements that comprise biomass will foster improved quantitative understanding of the links between chemical cycles and the physiology of organisms.

## Introduction

Ecological stoichiometry considers individuals as collections of chemical elements akin to a very large molecule. At the most fundamental level, ecological stoichiometry is the study of the sub-organismal mechanisms, and supra-organismal consequences of the principle of mass balance. It operates on the axiom that living entities are not a passive conduit of chemical supply, but rather actively regulating their elemental stoichiometry, referred to as elemental homeostasis (Sterner and Elser, 2002). Elemental homeostasis is the fulcrum for most stoichiometric models predicting processes at the level of the individual- (e.g., Frost et al., 2005), population- (e.g., Andersen et al., 2004), community- (e.g., Elser and Urabe, 1999), ecosystem- (e.g., Sterner et al., 1992), and global (e.g., Doney et al., 2009; Galbraith and Martiny, 2015) levels of organization. Indeed, without elemental homeostasis “ecological stoichiometry would be a dull subject” (Sterner and Elser, 2002).

**Figure 1:**
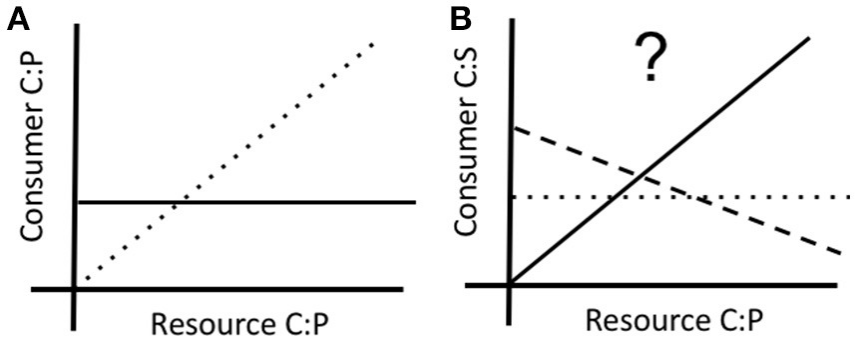
**(A)** Schematic representation of carbon: phosphorus (C:P) homeostasis in relation to supply C:P in homeostatic (solid line) and non-homeostatic (dotted line) consumers. **(B)** Hypothesized correlated shifts in carbon: sulfur (C:S) stoichiometry in the same consumer, showing no effect of P on S (dotted line), decrease in S under P limitation (dashed line), and increase in S under P-limitation (solid line).

At least two different approaches have been used to quantify the degree of elemental homeostasis (Sterner and Elser, 2002; Meunier et al., 2014). We quantify it using the slope of the log-log relationship between resource and consumer stoichiometry (Figure 1). Most stoichiometric models assume osmotrophs at the base of food webs exhibit relaxed stoichiometric homeostasis compared to phagotrophs occupying higher trophic levels. While this assumption has been a subject of debate and found to be of negligible relevance to stoichiometric models in consumers (Wang et al., 2012) the great diversity in the degree of elemental homeostasis (e.g., Frost et al., 2005; Scott et al., 2012; Godwin and Cotner, 2014, 2015; Meunier et al., 2014) remains largely unexplained. Such diversity is surprising because nutrient supply environments can impose strong selection on elemental quotas and consumption, which may be linked to stoichiometry. For example, Godwin and Cotner (2015) found that P content of isolating medium selected for strains of heterotrophic bacteria differing in P homeostasis and elemental quotas. The eco-evolutionary processes that maintain such substantially divergent phenotypes in natural populations is a central frontier in ecological stoichiometry.

Variation in genomic architecture has a major effect on physiological responses of both autotrophs (e.g., *Arabidopsis;* Misson et al., 2005) and heterotrophs (e.g., *Daphnia;* Roy Chowdhury et al., 2014) to changes in supply stoichiometry with important implications for homeostasis and fitness (e.g., Jeyasingh et al., 2009). Although, several informative loci for P use have been identified in crop plants (recently reviewed in van de Wiel et al., 2016), it is important to note that different genes and physiological pathways can underlie similar homeostatic and fitness outcomes among genotypes in autotrophs (*Glycine max;* Li et al., 2016) and heterotrophs (*Daphnia pulicaria;* Sherman et al., in review). This raises the possibility that genotypes exhibiting differing degrees of elemental homeostasis can vary in other traits. At the simplest level, we can think of elements that share similar properties that can be replaced when supply of one is limiting (e.g., substituting P-lipids with S-lipids under P limitation; Van Mooy et al., 2009; Bellinger et al., 2014). It is possible that a homeostatic genotype can maintain P content via efficient use of P, while a flexible genotype decreases P content, but increases S content to maintain basic cellular functions (Figure 1). As such, we need to understand coupled elemental quotas (such as P and S), as well as selection operating on such pathways for a complete understanding of the processes maintaining phosphorus homeostasis in populations.

Potential for correlated changes in homeostasis along multiple elemental axes is perhaps more apparent when one considers the complex physiological adjustments organisms make to maintain net anabolism in limiting conditions of elemental supply. Seminal studies in *E. coli* revealed the complex nature of responses to P limitation (Van Bogelen et al., 1996), involving differential expression of ~400 proteins orchestrating not only P use physiology, but also those involving other bulk and trace elements. Such complex physiological responses appear to be common. For example, studies in *Pseudovibrio* (Romano et al., 2015), *Saccharomyces* (Boer et al., 2010), *Chlamydomonas* (Moseley et al., 2006), *Arabidopsis* (Misson et al., 2005), and *Daphnia* (Jeyasingh et al., 2011), reveal that organisms differentially express a significant proportion of genes and metabolic pathways depending on P supply, often by several-fold. While several candidate P-stress response genes are up-regulated (e.g., P transporters, phosphatases; reviewed in Jeyasingh and Weider, 2007), so are several hundreds of other genes involved in a variety of pathways. Merchant and Helmann (2012) provided a comprehensive treatise on the diversity of microbial strategies to variation in elemental supply. An important message arising from this work is the fundamental interconnectedness of elements in biomass. In other words, acclimatory or adaptive responses to supply stoichiometry often involve changes in the physiological processing of many elements.

In this perspective, we ask whether such broad physiological changes in response to supply stoichiometry alters the entire suite of elements encompassing an individual. Defined as the mineral nutrient and trace element composition of an organism, the ionome represents all the elements of cellular and organismal systems (Salt et al., 2008). As such, the ionome is a dynamic network of elements that underlies the morphological, anatomical, and physiological state of an organism, which are ultimately controlled by the genome in response to the environment. We review evidence in the literature as well as analyze experimental data to illuminate the dynamic nature of the ionome, and discuss its implications for elemental homeostasis specifically, and the framework of ecological stoichiometry in general.

## Evidence in the literature

Ionomics is a relatively new field that has focused primarily on plants (see Huang and Salt, 2016 for a recent review). Ionomics was first employed to better understand the genomic architecture underlying mineral and trace element use in *Arabidopsis*, because studying the use of one element resulted in an incomplete picture of the genotype-to-phenotype map (Lahner et al., 2003). This approach has since been used as a low-cost, multi-proxy diagnostic tool in agronomy (Baxter et al., 2014) as well as medicine (Malinouski et al., 2014). Ionomics connects genetic potential and evolutionary history to growth, physiology and fitness in contemporary ecological conditions. Studies on both *Saccharomyces cerevisiae* (Eide et al., 2005; Yu et al., 2012) and *Arabidopsis thaliana* (Baxter et al., 2008) clearly show that both genetics and supply stoichiometry alter ionomes. Specifically, Eide et al. (2005) characterized the ionomes of over 4,000 yeast strains and found considerable variation in all 13 elements quantified, with both strong positive (e.g., P-Co) and negative (e.g., P-S) correlations under optimal growth conditions. Furthermore, they found that genotypes with mutations in similar functional categories (e.g., vacuolar, mitochondrial) showed similar ionomic signatures. Yu et al. (2012) utilized gene deletion and open reading frame overexpression collections of yeast (~5,000 strains) and found general patterns in the genomic basis of ionomic divergence. Mutations in genes involved in protein metabolism or transport had the largest impacts on the ionome, followed by changes in gene copy number. Baxter et al. (2008) found that *Arabidopsis* exhibited consistent ionomic patterns depending on supply stoichiometry such that the nature of nutrient stress could be predicted based on the ionome. While P content of leaves decreased under P limitation, there was considerable variation among genotypes, making P content a poor predictor of physiological status compared to a six element (As, B, Co, Cu, P, Zn) model, which included strong positive (e.g., P-Cu) and negative (e.g., P-Zn) correlations among elements. Considering combinations of elements as phenotypes, as opposed to considering single elements at a time, allows for greater sensitivity in identifying stoichiometric variation because of the fundamental interconnectedness among elements in biomass (Baxter, 2015).

An underappreciated component in understanding ionomes is likely to be transmembrane elemental transport systems, with transporters possessing multi-element specificity found to be more common than previously appreciated (Morrissey et al., 2009; Mitani-Ueno et al., 2011). As such, ionomic approaches are well suited to illuminate the complex physiological adjustments that organisms make in response to changes in supply stoichiometry. For example, P limitation increases the expression of several high-affinity phosphate transporters, which are also known to take up As (Muchhal et al., 1996) and thus explains increased As content in P-limited plants. Similarly, plants are known to scavenge metals such as Zn to minimize the formation of complexes with P (Misson et al., 2005) which could underlie the observed increase of Zn content under P limited conditions.

Evidence for ionome-wide shifts from the field are also available. *Synechococcus* cells collected from regions of the Sargasso Sea that vary in N and P supply exhibited several-fold cell quota differences in a variety of elements (e.g., Mn, Ni, Zn) (Twining et al., 2010). In addition, utilizing a global dataset, Loladze (2014) reported striking changes in the ionomes of C_3_ plants from four continents in response to elevated atmospheric CO_2_. In general, elevated CO_2_ significantly decreased not only N and P content, but also several other elements, including K, Ca, S, Mg, Fe, Zn, Cu, and Mn. These observations clearly indicate that supply stoichiometry alters entire suites of elements, beyond the commonly studied Redfield elements. Understanding the dynamics and regulation of these minor elements is important not only in understanding the ecology and evolution of microbes and plants, but as discussed by Loladze (2014), the variation in the composition of trace elements plays an important role in the nutrition of consumers, including humans (Myers et al., 2014).

## Experimental evidence

Are there patterns in ionomic architecture relevant to key parameters in stoichiometric theory such as homeostasis of Redfield elements? The staggering diversity of stoichiometric physiologies discovered among strains of heterotrophic bacteria inhabiting glacial lakes in northern U.S.A (Godwin and Cotner, 2015) provides an ideal testbed for answering such questions. We studied a subset of strains that were found to exhibit divergent homeostatic coefficients in terms of phosphorus (5 flexible, heterostoichs; 4 inflexible, homeostoichs) at two levels of P supply (C:P = 100 and 10,000; see Supplementary Material for methods). The nine strains used in this study represented three unique genera (*Brevundimonas, Flavobacterium*, and *Sphingomonas*) with three strains coming from each genus. All three of the *Brevundimonas* strains were characterized as flexible, whereas *Flavobacterium* and *Sphingomonas* each had two strains characterized as inflexible and one strain as flexible. Although we expected strong strain-specific responses, we generally predicted that homoestoichs should exhibit greater changes in other elements (e.g., S) between high and low P supply conditions compared to heterostoichs due to upregulation of compensating mechanisms. Each strain was originally isolated from lakes within Minnesota using either agar plates or dilution isolation as described previously (Godwin and Cotner, 2015).

**Figure 2:**
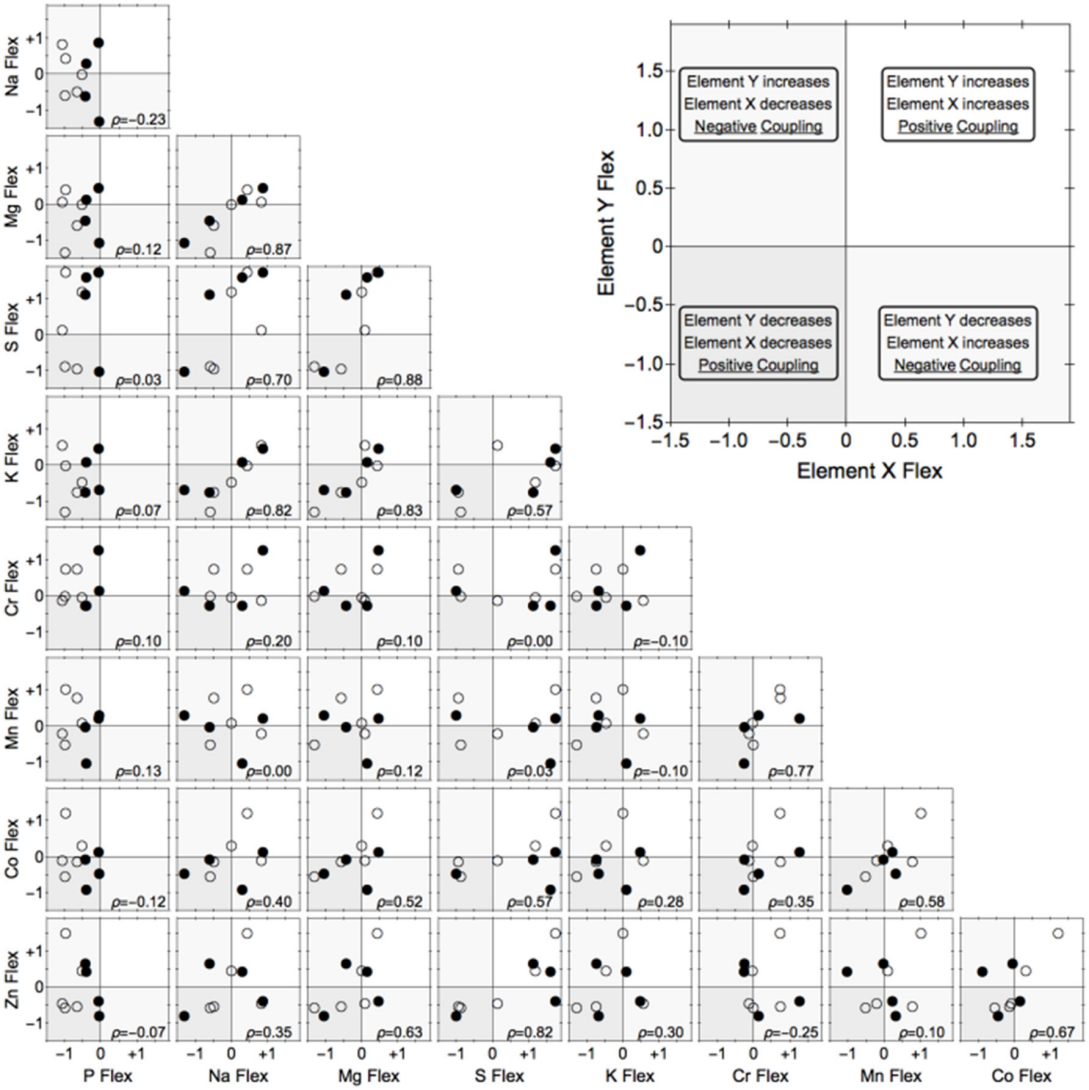
**Flexibility of nine elements in relation to each other in nine strains of freshwater heterotrophic bacteria**. We define flexibility as the log_10_[elemental content (mass/mass) at C:P of 10,000:1/ elemental content at C:P of 100:1]. Negative values represent a reduction in element quota under C:P = 10,000, zero means homeostasis, and positive values indicate that the quota increased under P-limitation. Symbols denote strains that are relatively flexible (open circles) or inflexible (closed circles) in phosphorus content. ρ = Spearman's rank coefficient.

A total of 25 elements were detected, of which nine (Co, Cr, K, Mg, Mn, Na, P, S, Zn) were present above detection limits in all samples and were used for further analyses. As expected, there was considerable strain-specific variation in biomass P content (Figure 2). Considerably more genotypic replicates are required to rigorously test for systematic differences among P homeostasis and correlated changes in the content of other elements.

Nevertheless, important trends were apparent in this dataset. We quantified the magnitude of elemental change in each strain and compared the differences between the two levels of homeostasis (hereto- vs. homeostoichs). Although considerable strain-specific responses preclude identification of any robust patterns, the response of the two homeostoich phenotypes appear to be distinct (Figure 2). Closer examination revealed that certain groups of elements were positively correlated with each other, namely Mg-Na-K, and to a lesser degree, Zn-S and Cr-Co-Mn (Figure 2). Of particular interest was how flexibility in P content related to flexibility of other elements (leftmost column in Figure 2). Consistent with previous studies (Godwin and Cotner, 2015), P content of all strains was lower under the C:P = 10,000 treatment. Interestingly, strains differed in the responses of other elements, with roughly half of the strains becoming more concentrated while the other half becoming less concentrated for the eight other elements. However, no systematic patterns with regard to the P homeostasis phenotype (flexible vs, inflexible) were apparent. We note that this preliminary result needs to be rigorously verified because the experiment had limitations (see Supplementary Material).

## Discussion

It is clear that ionomes are sensitive to both the external environment as well as the genomic composition. Whether such changes in the ionome are ecologically relevant is an important question worthy of attention by both empirical and theoretical practitioners of ecological stoichiometry. Studies with plants indicate that individual growth, even under strong P limitation is better predicted, not by P use efficiency alone, but by the uptake of a few other elements as well (Baxter et al., 2008). We posit that the correlated nature of elements in biomass predisposes organisms to tradeoffs in maintaining homeostasis of a particular element. If these tradeoffs occur, being homeostatic along one axis should be associated with changes in homeostasis along other elemental axes. At this point, we do not understand ionomes sufficiently to make robust predictions about what the most relevant trade-offs are. An understanding of the role and relevance of these other elemental axes, and the costs associated with the trade-offs among axes certainly is important to understanding the stoichiometry of organisms and ecological systems. The nature of such changes will depend on the material demands of biochemical pathways utilized to maintain homeostasis. Such an inclusive perspective of elemental homeostasis is required for understanding the diverse stoichiometric physiologies observed in both osmotrophic and phagotrophic populations (e.g., Frost et al., 2005; Godwin and Cotner, 2014, 2015; Meunier et al., 2014), and reflects the current state of evolutionary biology wherein the multifarious nature of selection is a prerequisite for understanding trait evolution (e.g., Kaeuffer et al., 2012).

As our understanding of nutrient limitation shifts from single nutrient models, to more complex, multiple nutrient models predicting co-limitation (e.g., Saito et al., 2008; Harpole et al., 2011; Bracken et al., 2015), the importance of attention to ionomic patterns in natural systems is magnified. Although there is a paucity of ionomic data in natural ecosystems, such data should reveal important patterns that could illuminate the mechanisms underlying co-limitation, which is increasingly common and should replace the Leibig paradigm (Kaspari and Powers, 2016). As such, simultaneous limitation of multiple elements may be strong sources of selection structuring populations with important implications for contemporary nutrient budgets. Nevertheless, it is unlikely that all of the 25-odd elements represented in biology will impart the same magnitude of selection or ecological significance. Although an organism should acquire all elements from the environment, some elements (e.g., copper) can be recycled within the organism quite efficiently that it may not need to be constantly acquired from the environment (Nose et al., 2006), while others (e.g., phosphorus) are excreted as a byproduct of metabolic processes and need to be constantly acquired from the environment. However, if P and Cu are coupled, then the evolutionary and ecological importance of Cu is amplified. Thus, focusing on correlations among elements may be a particularly informative approach.

Ionomic data from both the literature and our experiment reveal several correlations, although the functional basis for such correlations appears to be more complex than what can be predicted by linkages based on cellular physiology of element processing. For example, sodium dependent phosphate uptake by cells is a well-established mechanism (discussed in the context of ecological stoichiometry in Jeyasingh and Weider, 2007), yet sodium and phosphorus do not appear to be correlated at the ionome level. Clearly, much more remains to be understood about the complex processes underlying such patterns. For example, Malinouski et al. (2014) studied HeLa cell lines to characterize mechanisms that regulate trace elements by performing a genome-wide siRNA/ionomics screen to identify the major pathways. They analyzed a total of 21,360 human gene knockdowns for changes in trace elements in HeLa cells and detected many known genes involved in transport and regulation of trace elements while also identifying several novel genes that regulate the processing of trace elements. As such, the mechanisms underlying correlations among elements in an ionome is difficult, and perhaps of little ecological relevance. However, general patterns in correlations among elements at the level of the ionome will have important ecological ramifications. Discovering such patterns and associated ecological implications should be viewed as a central challenge.

Genotype-specific effects common in ionomic studies discussed above are similar to discoveries about intraspecific variation in biomass C:N:P stoichiometries (e.g., Bertram et al., 2008; Goos et al., 2014; Downs et al., 2016) which are shaped by selection (e.g., El-Sabaawi et al., 2012; Tobler et al., 2016), and generate discernable patterns at higher levels of organization (e.g., Elser et al., 2000). While genetic recombination can produce endless varieties of biota, organismal evolution is bounded by principles of physics and chemistry (Williams and Frausto da Silva, 2006). Ecological stoichiometry, by virtue of abstracting such complexity, has unraveled general patterns linking elements such as phosphorus with fitness-relevant traits and subsequent ecological consequences. The focus on only three of the ~25 elements represented in biology is limited, however, and perhaps has misrepresented both patterns and processes in ecological stoichiometry. Whether, and to what extent, predictions of stoichiometric models are enhanced, similar to those predicting individual growth (e.g., Baxter et al., 2008), by inclusion of the entire suite of elements remains to be seen. Advances in low-cost, high throughput elemental analyses already enable an ionomic view of ecological stoichiometry, and such data will be required to make sense of central parameters in stoichiometric models in light of genomic information, and perhaps also metagenomic data using meta-ionomics, for a more comprehensive genes-to-ecosystems picture of the biosphere.

## Author contributions

All authors made equal, substantial contributions toward the creation of this manuscript, and approve its publication.

### Conflict of interest statement

The authors declare that the research was conducted in the absence of any commercial or financial relationships that could be construed as a potential conflict of interest.
