# Supplementary material for: Ecological Stoichiometry beyond Redfield: An Ionomic Perspective on Elemental Homeostasis
Source: Front Microbiol. 2017 Apr 25;8:722. doi: 10.3389/fmicb.2017.00722 (PMC5403914; doi:10.3389/fmicb.2017.00722)
Supplement: Supplementary file 1 [file Table1.docx]

***Supplementary Material***

*Ecological stoichiometry beyond Redfield: An ionomic perspective on elemental homeostasis*

*Punidan D. Jeyasingh, Jared M. Goos, Seth K. Thompson, Casey M. Godwin, James B. Cotner *Correspondence: Corresponding Author: puni.jeyasingh@okstate.edu

**1** **Experimental and analytical methods**

All strains were grown as batch cultures in defined media with either glucose or acetate as the sole C-source (BMM; Tanner 2002) on a shaking incubator (120 rpm) at 22℃. All cultures were incubated until biomass accumulation caused visible turbidity (3-21 days depending on strain and media type), at which point cells were collected onto 0.45 µm nominal pore-size cellulose acetate membrane filters (Whatman OE 67, GE Life Sciences) using low pressure vacuum filtration. All filters were dried (60℃) and microbial biomass collected on the filter was measured by mass pre/post filtration. Filters were analyzed for 25 elements in total using an inductively-coupled plasma optical emission spectrometer (ICP-OES; Thermo Scientific iCAP 7400, Waltham, MA, USA) after digestion (24h in 70% trace metal grade HNO_3_) in 15 mL metal-free polypropylene tubes. Validation and calibration of the ICP-OES analysis was performed by using aqueous multi-element external standard reference solutions (CPI International, Santa Rosa, CA, USA). Additionally, we used an in-line internal standard of yttrium (CPI International, Santa Rosa, CA, USA) to correct for instrument drift or potential matrix effects. Elemental concentrations were excluded from further analysis if measured concentrations were within the range of the standard deviation of our blank controls, indicating that those concentrations are close to or below the detection limit of the instrument.

**2** **Limitations of the experiment**

In addition to low genotypic replicates, coarse resolution of supply stoichiometry and batch culturing, as well as differences in carbon source (5 strains; 3 hetero- and 2 homeostoichs were grown with acetate as the C source in the C:P= 10,000 treatment because they did not grow to sufficient biomass in glucose) are key limitations of this experiment. Furthermore, studies in both heterotrophic bacteria (Godwin et al. 2016) and phytoplankton (Goldman et al. 1979; Hillebrand et al. 2013) have shown that growth rate and resource imbalance interact to affect biomass stoichiometry. Moreover, a recent experiment by Garcia et al (2016) found that increasing the growth rate of *Synechococcus* in chemostats caused the cells to become larger, and that this increase in size was associated with an increase in the quota of non-limiting elements. Nutrient limitation can also change microbial size (Phillips et al., In review), complicating interpretation of stoichiometric responses. Future studies should pay close attention to separating the effects of nutrient limitation from that of growth and other traits such as size, on the ionome for a clearer picture of elemental linkages in organisms, and how such links change in relation to supply stoichiometry.

**References cited:**

Garcia, N.S., Bonachela, J.A., and Martiny, A.C. (2016). Interactions between growth-dependent changes in cell size, nutrient supply and cellular elemental stoichiometry of marine Synechococcus. *Isme Journal* 10(11)**,** 2715-2724. doi: 10.1038/ismej.2016.50.

Godwin, C.M., Whitaker, E.A., and Cotner, J.B. (2016). Growth rate and resource imbalance interactively control biomass stoichiometry and elemental quotas of aquatic bacteria. *Ecology*. doi: 10.1002/ecy.1705.

Goldman, J.C., Mccarthy, J.J., and Peavey, D.G. (1979). Growth-Rate Influence on the Chemical Composition of Phytoplankton in Oceanic Waters. *Nature* 279(5710)**,** 210-215. doi: DOI 10.1038/279210a0.

Hillebrand, H., Steinert, G., Boersma, M., Malzahn, A., Meunier, C.L., Plum, C., et al. (2013). Goldman revisited: Faster-growing phytoplankton has lower N : P and lower stoichiometric flexibility. *Limnology and Oceanography* 58(6)**,** 2076-2088. doi: 10.4319/lo.2013.58.6.2076.

Phillips, K. N., Godwin, C. M., & Cotner, J. B. (In review). Sensitivity of heterotrophic bacteria in aquatic systems to nutrient imbalances and warming. *Frontiers in Microbiology*.

Tanner, R. (2002). "Cultivation of bacteria and fungi," in *Manual of Environmental Microbiology,* ed. G.K. C. Hurst, M. Mcinerney, L. Stetzenbach, and M. Walter (Washington, DC: ASM Press), 62-70.
